# Healthful Contemplations

**Published:** 1856-04

**Authors:** 


### HEALTHFUL CONTEMPLATIONS.

MORAL Philosophers live longer than any other class of men, showing the influence which the prevailing state of the mind has on the human health. There is something delightfully luscious and soul-feeding in the contemplation of a new idea, which, with resistless power presses home upon the heart the conviction of the goodness of God, leaving, as it often does, a feeling of subdued happiness, in which we delightfully revel for a long time after. We hold that contemplations like these have a sanitory influence on mind and body, which has not been duly estimated. Hence we hope to throw out such thoughts to the people from time to time, as will be likely to open up a fountain of health, which most benefits those who oftenest repair to it. We do not promise that these ideas shall be new to all of our readers, but they will be new to some.

If you take up a piece of slate or a common stone, you will often see a yellow shining crystal like brass. The first impression on the unscientific is, that it is gold, hence perhaps the common saying "*all is not gold that glitters.*" These yellow shining crystals are formed of Iron and Sulphur, and are called *Iron Pyrites:* and contain Arsenic, yielding, if thrown into the fire, Arseneous acid, the fumes of which are a certain and deadly poison. But although this corroding poison is frequently found combined with *Iron Pyrites*, in *one* situation it never is thus combined, to any hurtful extent; that is, in *Iron Pyrites* as found among the *Coal formations* of the world, no compounds of Arsenic have ever been discovered. Suppose for a moment the *Iron Pyrites* found in the coal formations, were like the *Iron Pyrites* of other localities, the simple result would be, according to our present knowledge, that the coal mines of the world would be useless, because the fumes which the coal would give out when placed in the grate, would be destructive of human life, and no one would dare to employ it for domestic purposes; thus, at once, a mine of wealth, richer by far than the gold of all the globe, would be closed up, as worse than worthless. How kindly wise then, is that *Great Being* who made all worlds, in adapting his creations to the safety and happiness of us his children. In the case before us, by withholding a single constituent in a formation under one set of circumstances, which is present in other circumstances, he converts what otherwise would have been a curse, if used, into one of the greatest comforts and blessings of civilized life. Further, if Arsenic was found only *sometimes* in the Iron Pyrites of the coal fields, its destructive effects would alarm all away from its employment under any circumstances; but the broad fact stands out in uniform distinctness, that although *Iron Pyrites* is found in almost every rock, stratified and unstratified, and when thus found, liable to contain Arsenic, yet, when found in the coal with which we warm our apartments, and cook our food, and light our streets, and propel our steamships, and drive our machineries, and work our locomotives, in that coal, it is never found in those formations, it never exists!

Let us then, as a means of health, feed more on the *beneficences of our Creator;* it is a food which strengthens the mind, elevates the soul, enlarges the heart, and leads the whole man upward and onward by a pathway full of light and flowers, and sunshine, a pathway smooth, and safe, and sure, where no snare is ever set, where lurking dangers never come, whose beginning is in a world of trial, whose ending is in the bosom of God!

